# Supplementary material for: Characterization of carbon metabolism in a highly adhesive bacterium Acinetobacter sp. Tol 5 capable of assimilating diverse hydrocarbons and aromatic compounds
Source: Front Microbiol. 2026 Aug 31;17:1919423. doi: 10.3389/fmicb.2026.1919423 (PMC13572102; doi:10.3389/fmicb.2026.1919423)
Supplement: Supplementary file 1 [file Data_Sheet_1.PDF]

Supplementary Material

Supplementary Figures

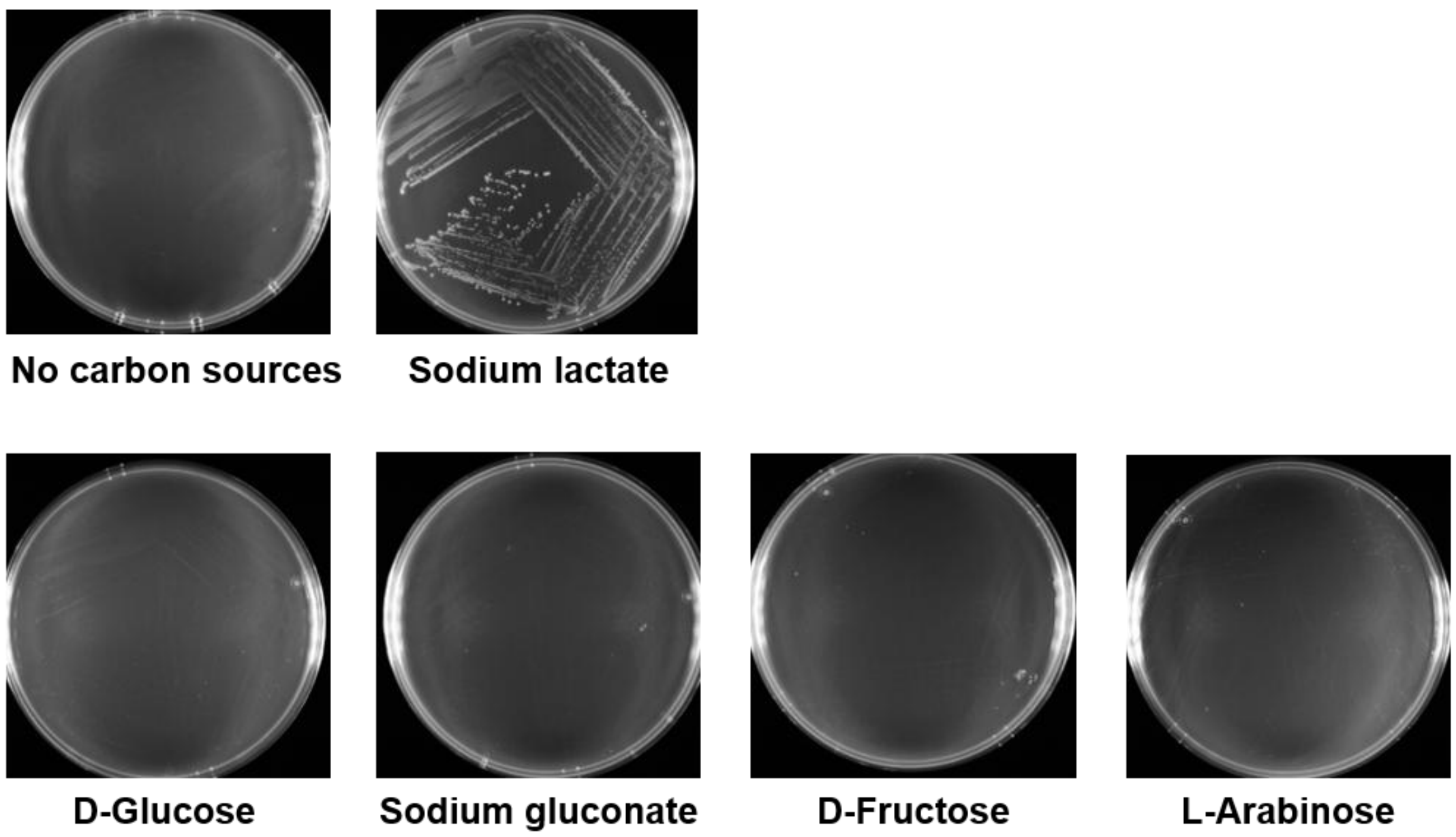

Supplementary Figure 1. Growth of Tol 5 Δ*ataA* on sugars. The Tol 5 Δ*ataA* was streaked on BS agar plates supplemented with sodium lactate, D-glucose, sodium gluconate, D-fructose, or L-arabinose as the sole carbon source and incubated for 5 days. Images are representative of at least two independent biological replicates with similar results.

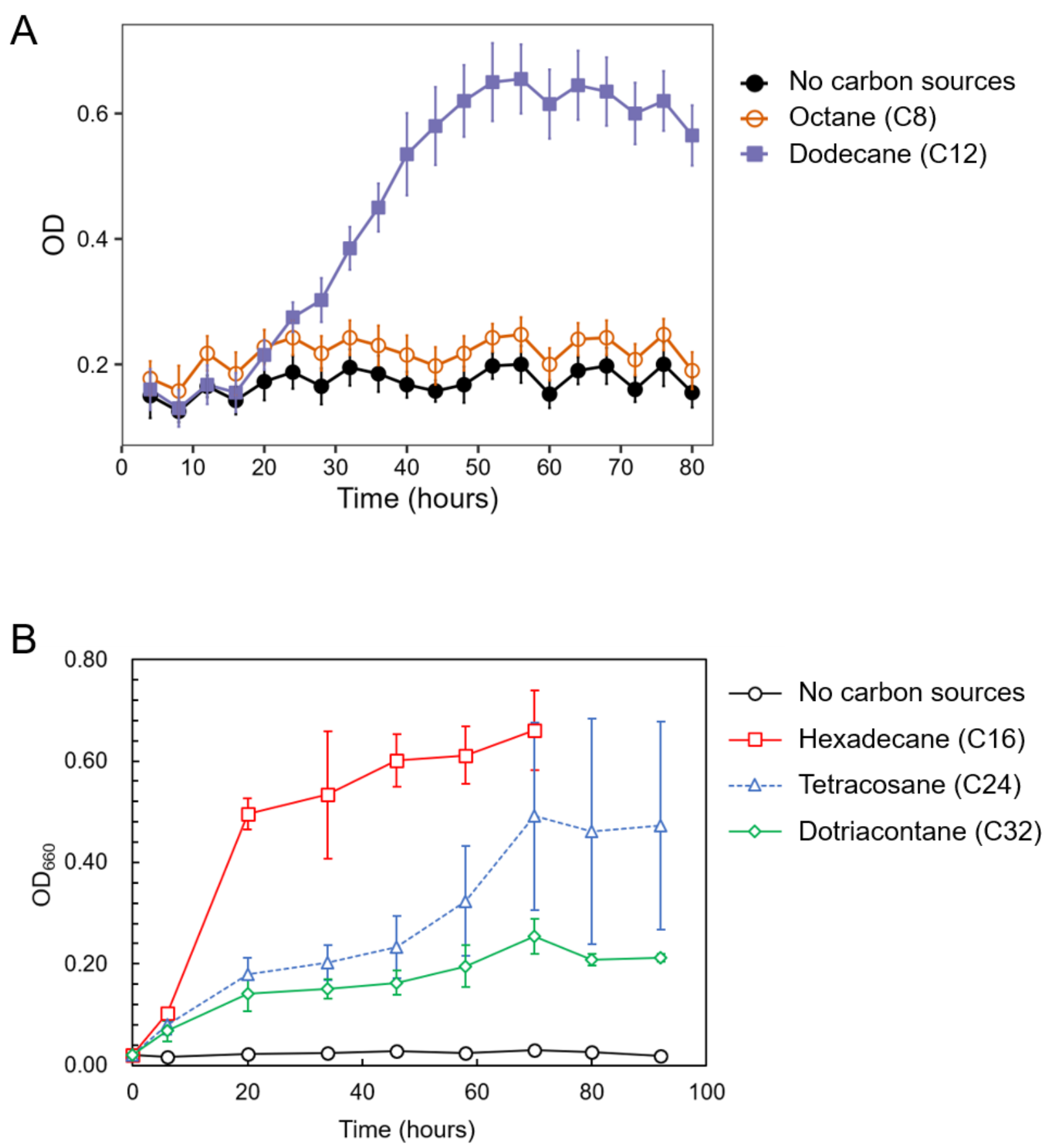

Supplementary Figure 2. Growth of Tol 5 on *n*-alkanes of different chain lengths. The Tol 5 Δ*ataA* mutant was grown in BS medium with each *n*-alkane as the sole carbon source. (**A**) Growth on octane (C8) and dodecane (C12), measured in glass tubes with automatic OD monitoring using the OD-monitor C&T system. (**B**) Growth on hexadecane (C16), tetracosane (C24), and dotriacontane (C32), measured in flasks by sampling and measuring OD_{660} with the UV–Vis spectrophotometer. All data are presented as the means ± SD (biological replicates n = 3).

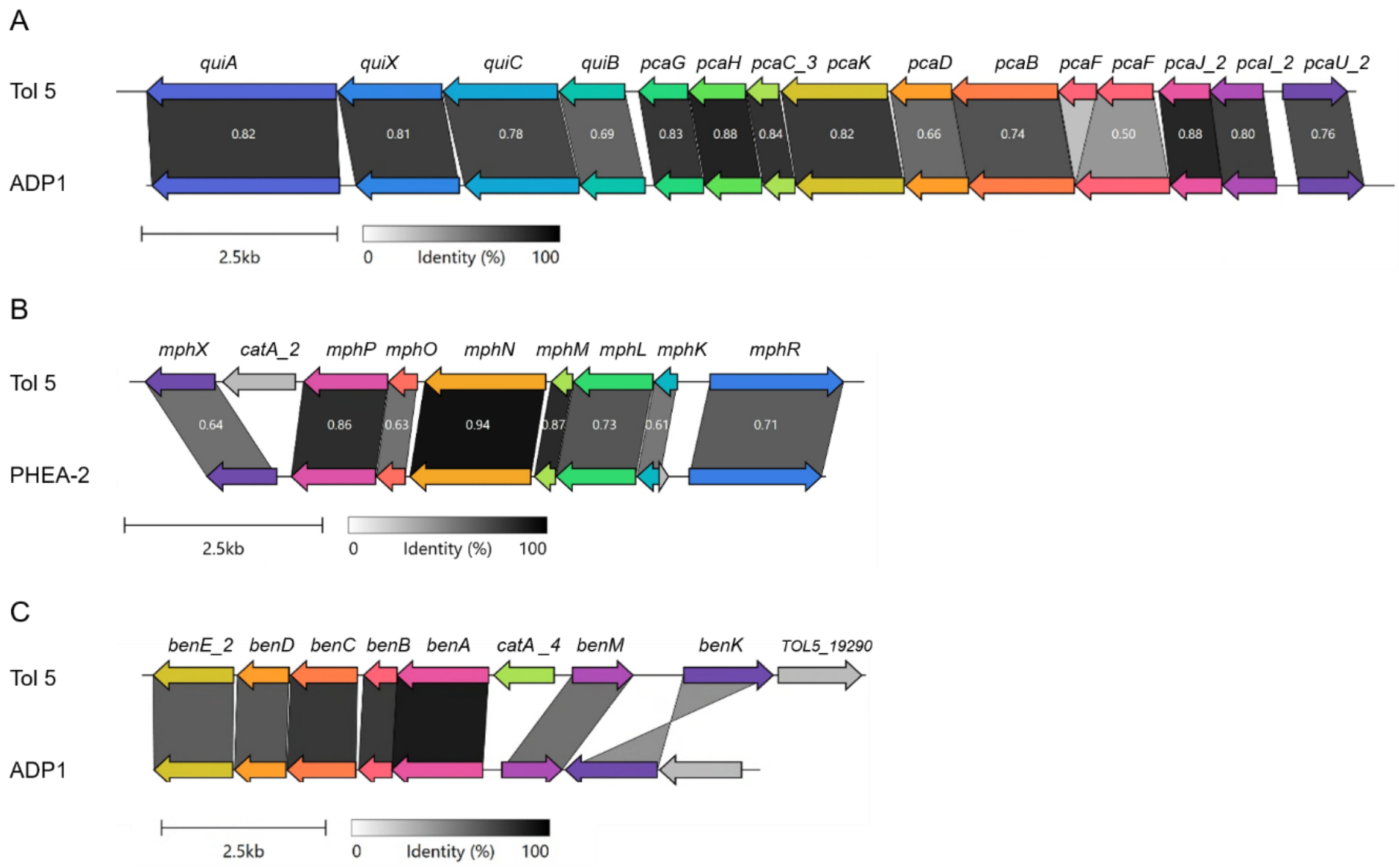

Supplementary Figure 3. Organization of aromatic degradation gene clusters in Tol 5 compared with related *Acinetobacter* species. Schematics represent the genomic organization of the *pca-qui* (**A**), *mph* (**B**), and *ben* (**C**) gene clusters of Tol 5, aligned using clinker with the corresponding clusters of *A. baylyi ADP1* (accession: CR543861.1) or *A. calcoaceticus* PHEA-2 (accession: CP002177.1). Arrows indicate predicted coding sequences and their transcriptional orientation. Homologous genes are connected between clusters.

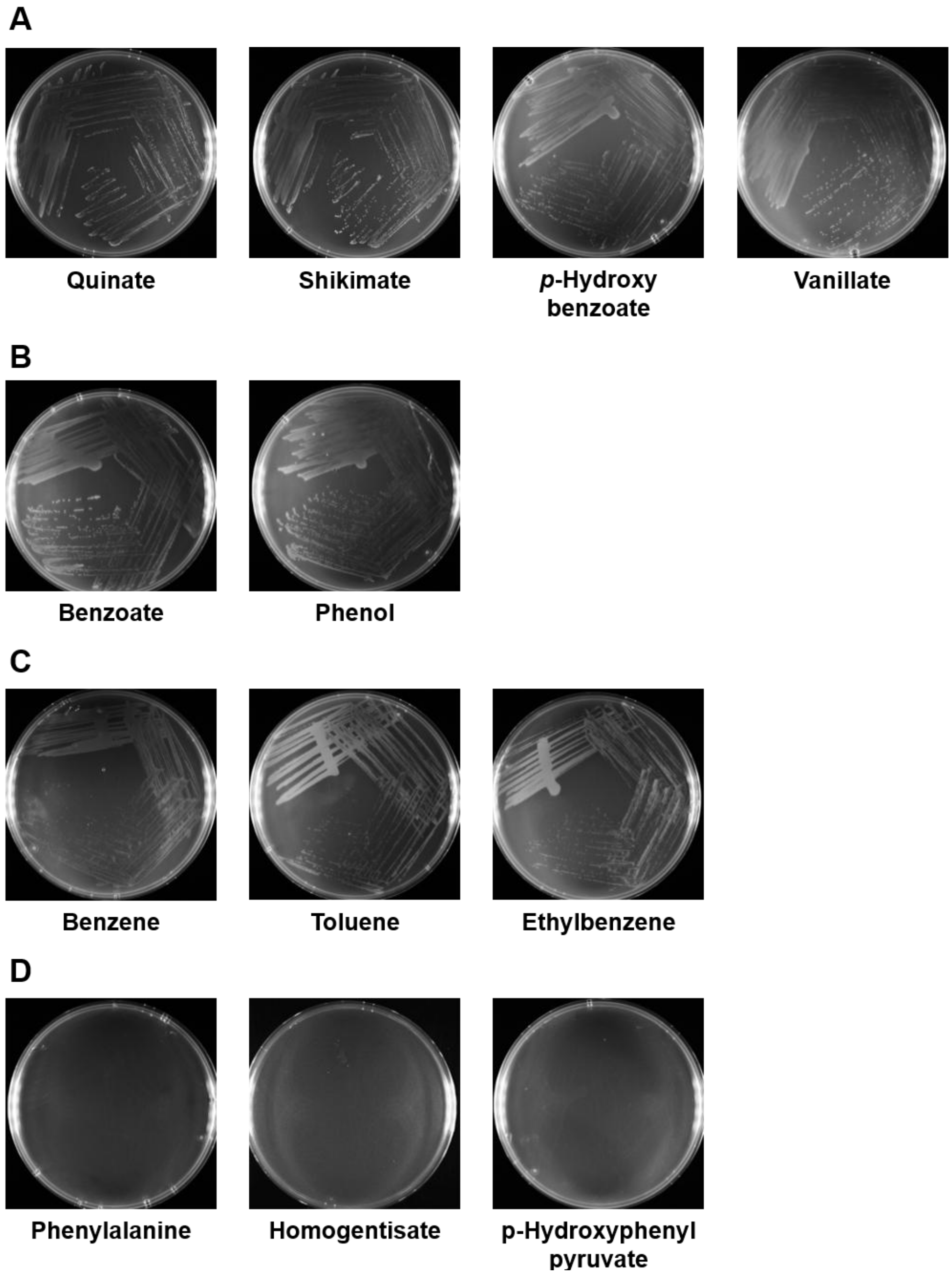

Supplementary Figure 4. Growth of Tol 5 on aromatic compounds on agar plates. The Tol 5 ΔataA mutant was streaked on BS agar plates supplemented with aromatic compound as the sole carbon source and incubated for 5 days. Plates were sealed in AnaeroPack pouches and incubated for 5 days. Volatile substrates were supplied in the vapor phase. (**A**) Substrates related to the PCA pathway: quinate, shikimate, *p*-hydroxybenzoate, and vanillate. (**B**) Substrates related to the CAT pathway: phenol and benzoate. (**C**) Substrates related to the TOD pathway: toluene, benzene, and ethylbenzene. (**D**) Substrates related to aromatic amino acid metabolism: L-phenylalanine, homogentisate, and *p*-hydroxyphenylpyruvate. Images are representative of at least two independent biological replicates with similar results.

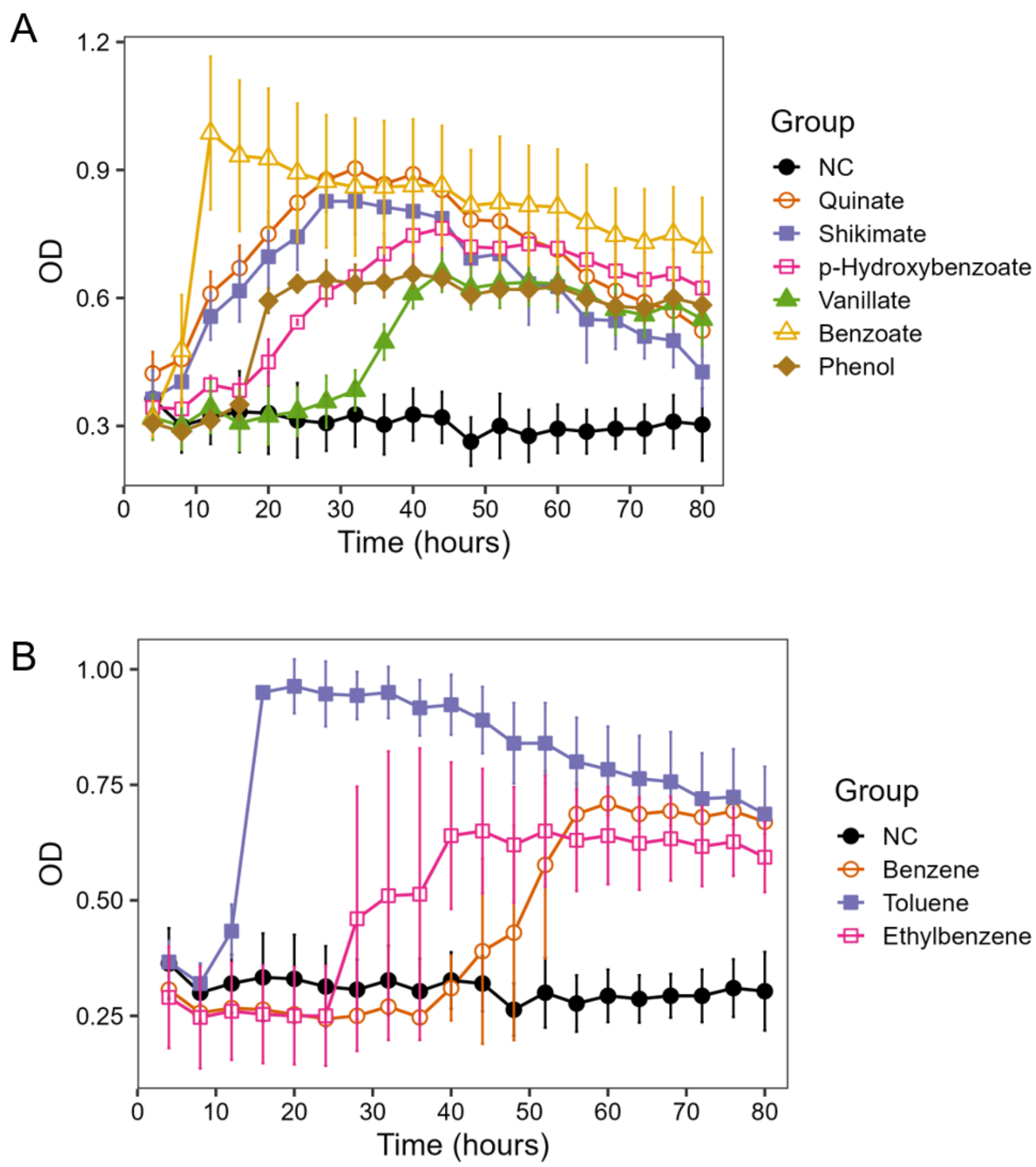

Supplementary Figure 5. Growth of Tol 5 on aromatic substrates. The Tol 5 Δ*ataA* mutant was grown in BS medium with each aromatic substrate as the sole carbon source, and the OD was automatically monitored in glass tubes using the OD-monitor C&T system. (**A**) Substrates funneled through the PCA and CAT pathways: quinate, shikimate, *p*-hydroxybenzoate, vanillate, benzoate, and phenol. (**B**) Substrates degraded via the TOD pathway: benzene, toluene, and ethylbenzene. NC, no carbon source control. Data are presented as the means ± SD (n = 3 biological replicates).

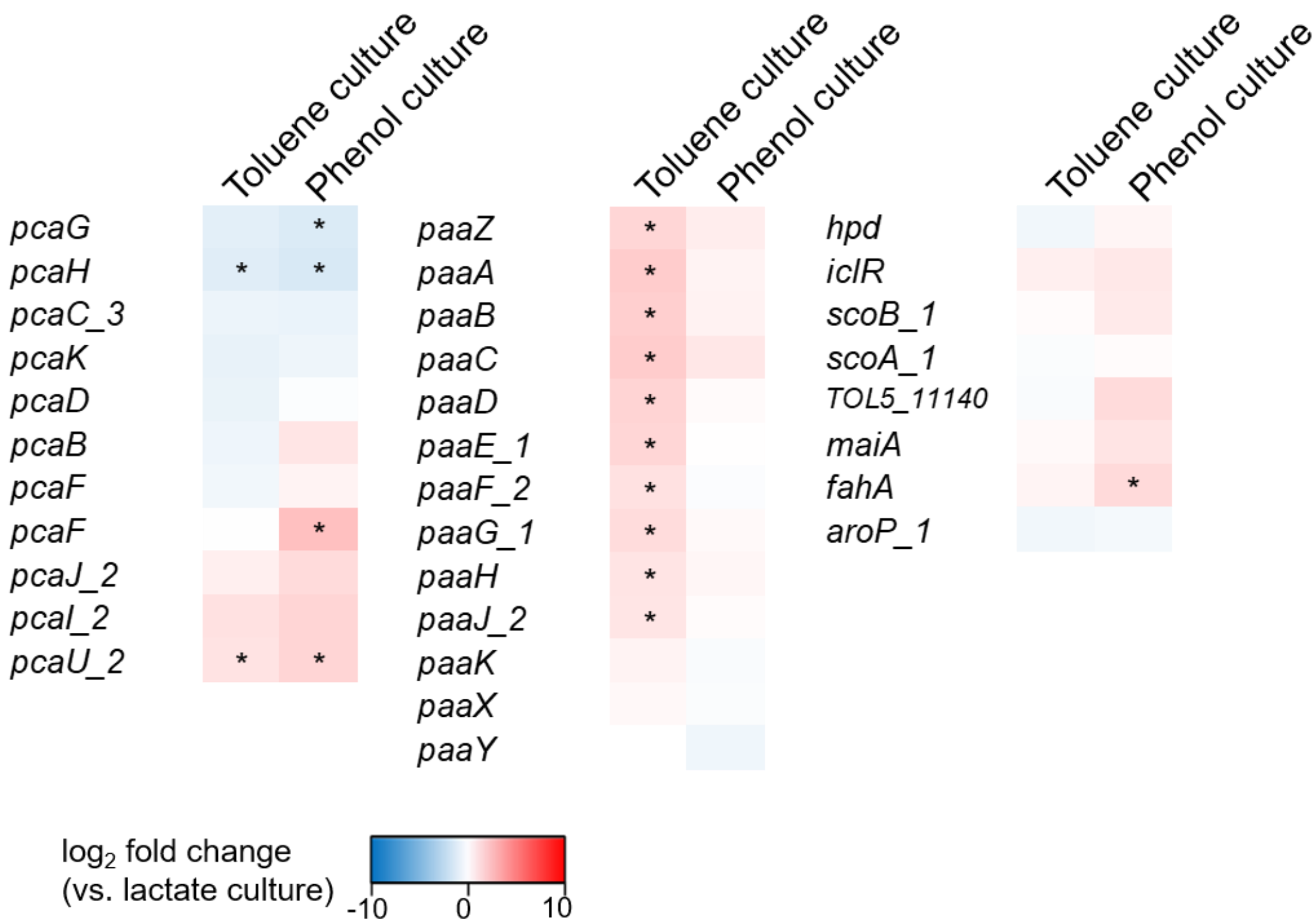

Supplementary Figure 6. Heat maps showing the average $\log_2$ FC of the genes involved in the degradation of aromatic compounds (*pca*, *paa*, and *hmg* genes). The color gradient indicates the magnitude of the $\log_2$ FC values, with the deepest red representing ≥ 10 and the deepest blue representing ≤ -10. Asterisks (*) denote differentially expressed genes defined by $|\log_2 \text{FC}| > 1$, $\log_2$ CPM > 3, and FDR < 0.01.

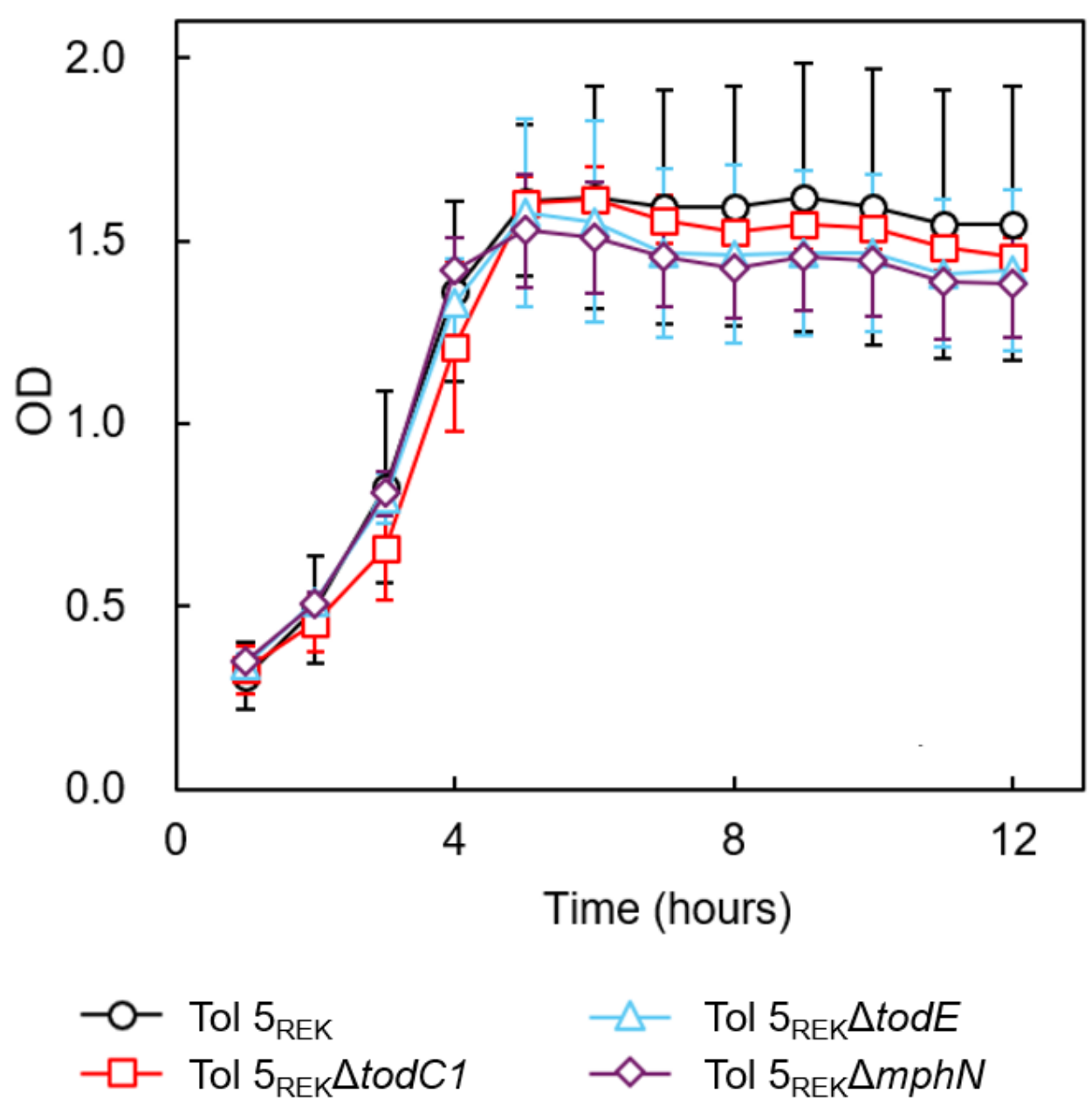

Supplementary Figure 7. Growth of gene disruption mutants on sodium lactate. Tol 5_{REK} (circles), Tol $5_{REK}\Delta$*todC1* (squares), Tol $5_{REK}\Delta$*todE* (triangles), and Tol $5_{REK}\Delta$*mphN* (diamonds) were inoculated to BS medium containing lactate at a carbon equivalent concentration of 1.4×10^{-2} mol/L, and OD was monitored using the OD-monitorC&T system. Data are presented as means ± SD (biological replicates n = 3).

Supplemental Tables

Supplementary Table 1. Strains and plasmids used in this study.

Strain	Description	Reference
Acinetobacter sp.		
Tol 5	Wild type strain	(Hori et al., 2001)
Tol 5 Δ*ataA*	*ataA* deletion mutant of Tol 5	(Ishikawa et al., 2012)
Tol 5_{REK}	Restriction enzyme-encoding genes and *ataA* knockout mutant of Tol 5, REK123Δ*ataA*	(Ishikawa and Hori, 2024)
Tol 5 $_{REK}$ Δ*todC1*	*todC1* gene knockout mutant of Tol 5_{REK}	(Yoshimoto et al., 2025)
Tol 5 $_{REK}$ Δ*todE*	*todE* gene knockout mutant of Tol 5_{REK}	This study
Tol 5_{REK} Δ*mphN*	*mphN* gene knockout mutant of Tol 5_{REK}	This study
Escherichia coli		
DH5α	Host for routine cloning	Purchased from Takara Bio (Shiga, Japan)
Plasmid		
pBECAb-apr	For gene knockout	(Wang et al., 2019)
pBECAb-apr-todE	For *todE* gene knockout	This study
pBECAb-apr-mphN	For *mphN* gene knockout	This study

Supplementary Table 2. Oligo DNAs used in this study.

Primer name	Sequence (5' to 3')
TodE-sgRNA-Fw	CTAAGCGGTCTCTTAGTAATCGAGTTGAGGTGTATTAGTTTGGAGACCCGTAGC
TodE-sgRNA-Rv	GCTACGGGTCTCCAAACTAATACACCTCAACTCGATTACTAAGAGACCGCTTAG
MphN-sgRNA-Fw	TAGTAAACTCCCAGTCCAGATCAC
MphN-sgRNA-Rv	AAACGTGATCTGGACTGGGAGTTT

Supplementary Table 3. Functional predictions of genes in Tol 5 based on multiple databases. (Excel file)

Supplementary Table 4. Genes predicted to be involved in the metabolism in Tol 5. (Excel file)

Supplementary Table 5. Information about the RNA-seq data used in this study

Substrates	Total reads (raw FASTQ)	Total reads after fastp filtering	Total mapped reads	Mapping rate (%)
Lactate	12813627	12653268	11336668	89.6
	12421215	12298185	10803072	87.8
	12339378	12221016	11190080	91.6
Ethanol	12814610	12700050	11516082	90.7
	11870987	11766340	10751266	91.4
	12887700	12771057	11582603	90.7
Hexadecane	11487878	11231169	9913352	88.3
	11814155	11698621	10507217	89.8
	12704470	12510943	11381040	91.0
Toluene	12476544	12235527	11115768	90.8
	11862557	11754526	10750795	91.5
	10735004	10650101	9637121	90.5
Phenol	11282222	11144094	10095402	90.6
	10571014	10447506	9312043	89.1
	10544517	10445993	9367032	89.7

Supplementary Table 6. Comparison of gene expression in Tol 5 grown on various carbon sources relative to lactate. (Excel file)

Supplementary Table 7. Differentially expressed genes in Tol 5 grown on various carbon sources relative to lactate. (Excel file)

References

Hori, K., Yamashita, S., Ishii, S.i., Kitagawa, M., Tanji, Y., and Unno, H. (2001). Isolation, Characterization and Application to Off-Gas Treatment of Toluene-Degrading Bacteria. *Journal of Chemical Engineering of Japan* 34(9)**,** 1120-1126.

Ishikawa, M., and Hori, K. (2024). The elimination of two restriction enzyme genes allows for electroporation-based transformation and CRISPR-Cas9-based base editing in the non-competent Gram-negative bacterium *Acinetobacter* sp. Tol 5. *Applied and Environmental Microbiology* 90(6)**,** e00400-00424. doi: 10.1128/aem.00400-24.

Ishikawa, M., Nakatani, H., and Hori, K. (2012). AtaA, a New Member of the Trimeric Autotransporter Adhesins from *Acinetobacter* sp. Tol 5 Mediating High Adhesiveness to Various Abiotic Surfaces. *PLOS ONE* 7(11)**,** e48830. doi: 10.1371/journal.pone.0048830.

Wang, Y., Wang, Z., Chen, Y., Hua, X., Yu, Y., and Ji, Q. (2019). A Highly Efficient CRISPR-Cas9-Based Genome Engineering Platform in *Acinetobacter baumannii* to Understand the H_2O_2-Sensing Mechanism of OxyR. *Cell Chemical Biology* 26(12)**,** 1732-1742.e1735. doi: https://doi.org/10.1016/j.chembiol.2019.09.003.

Yoshimoto, S., Hattori, M., Inoue, S., Mori, S., Ohara, Y., and Hori, K. (2025). Identification of toluene degradation genes in *Acinetobacter* sp. Tol 5. *Journal of Bioscience and Bioengineering* 140**,** 284-289. doi: 10.1101/2025.05.12.653411.
